# Choosing Less Harmful Alternatives: The Ethics of Harm Reduction in Emerging Technologies

**DOI:** 10.1007/s11948-025-00570-z

**Published:** 2025-12-04

**Authors:** Cody Turner

**Affiliations:** Department of Philosophy, Bentley University, Waltham, MA USA

**Keywords:** Harm reduction, Emerging technologies, Functional equivalence, Technology ethics, Food ethics, Transportation ethics

## Abstract

When are we obligated to choose less harmful alternatives to existing practices? This article addresses this deceptively simple question by developing the *Principle of Choosing Less Harmful Alternatives (PCLHA)*, which holds that it is morally wrong to continue to engage in a practice that causes harm when an affordable, accessible, functionally equivalent, less harmful alternative exists. While PCLHA pertains to any practice for which its conditions are met, the principle is particularly valuable for contingent (rather than intrinsic) wrongs, delineating a sufficient condition for when technological or social progress renders once-permissible practices impermissible. I pressure-test PCLHA by applying it to emerging technologies across several domains, including food ethics (cultivated meat), transportation ethics (autonomous vehicles), and leisure ethics (virtual reality tourism and sex bots). Through these applications, I demonstrate that the principle faces two opposing challenges: it appears simultaneously too weak, by not requiring switches to less harmful alternatives when they fall short of full functional equivalence (e.g., allowing factory farming despite massive harm), and too strong by requiring switches to alternatives in cases where this feels intuitively overreaching (e.g., mandating virtual reality tourism). I address these challenges via complementary modifications to PCLHA: a ‘Sliding Scale Modification’ that allows the required degree of functional equivalence to vary with harm severity, and a ‘Threshold of Harm Qualification’ that limits PCLHA’s application to cases of significant harm.

## Introduction

When are we obligated to choose less harmful alternatives to prevailing practices? Some practices are *intrinsically* wrong in that they are wrong regardless of the existence of alternatives (e.g., torture for amusement). Other practices are *contingently* wrong in that they only become impermissible when less harmful alternatives exist. Performing surgery on people without anesthesia is a plausible example of such a practice: it only became wrong once ether and chloroform were made available around the 1840s. For practices that are contingently wrong, the central question concerns the conditions under which the moral valence of the practice shifts. This article addresses this question by developing the *Principle of Choosing Less Harmful Alternatives (PCLHA)*. PCLHA holds that it is morally wrong to continue to engage in a practice that causes harm when an affordable, accessible, functionally equivalent, less harmful alternative exists. While PCLHA pertains to any practice for which its conditions are met, the principle is particularly valuable for contingent wrongs, functioning as a moral trigger point that delineates when technological or social progress renders once-permissible practices impermissible.

PCLHA is not meant to be a controversial normative principle. To the contrary, I propose it as a sufficient condition for moral wrongness that every reasonable person and normative theory should accept. The principle is intuitively obvious because it sets such a low bar for moral obligation. The numerous qualifications built into PCLHA effectively screen out cases where switching practices could be perceived to be unfairly demanding on an individual or group, whether because the less harmful alternative is too expensive, too scarce, or sacrifices too much functionality.

Much of the article’s value lies in showing that even obvious normative principles necessitate careful conceptual analysis in order to be actionable. Several questions immediately arise when applying PCLHA to concrete cases. What exactly does it mean for an alternative to be ‘functionally equivalent’ to an existing practice? How significant must both the harm from the existing practice and the reduction in harm from the alternative be to make continuing with the existing practice morally wrong? How does one adjudicate tradeoffs between functionality and harm reduction among competing practices? What about when there are tradeoffs between functions themselves?

To illuminate these questions and pressure-test PCLHA, I apply the principle to the domains of food ethics, transportation ethics, and leisure ethics. Through this analysis, I show that PCLHA faces the seemingly paradoxical challenge of being simultaneously too weak and too strong. On the one hand, the principle can be criticized for being too weak or insufficiently demanding because it does not require switching to less harmful alternatives when they fall short of full functional equivalence (even in cases where the harm reduction would be massive, like the factory farming example discussed in the third section). On the other hand, one might object that the principle is too strong because it calls for a switch to less harmful alternatives in cases where this feels intuitively overreaching (such as the VR tourism and sex bot examples discussed in the fourth section). 

I respond to the ‘too weak’ objection by adding a ‘Sliding Scale Modification’ to PCLHA that allows for varying degrees of required functional equivalence based on harm severity, and the ‘too strong’ objection with a ‘Threshold of Harm Qualification’ that limits PCLHA’s application to cases of significant harm. These modifications to the principle are complementary: the threshold of harm qualification determines when PCLHA applies at all, whereas the sliding scale modification specifies how strictly to interpret the functional equivalence condition once it does apply. Notably, while this article centers on PCLHA, there are many closely related harm reduction principles in conceptual space.

Traditional harm reduction approaches focus on mitigating harms from intractable, often criminalized practices that persist regardless of legal status (e.g., injection drug use). PCLHA, by contrast, pertains to harmful practices that can be replaced, not merely made safer. Dea (2020) observes that the concept of ‘harm reduction’ has received surprisingly little philosophical attention, despite extensive philosophical literature on the notion of ‘harm’ (e.g., Kahane & Savulescu, 2012; Purves, 2019). Dea calls for a more unified, recognizable philosophy of harm reduction, one that extends existing harm reduction principles to novel domains and formulates novel harm reduction principles. This article answers that call by presenting and evaluating PCLHA in the context of emerging technologies. The structure of the article is as follows. Section “The Principle of Choosing Less Harmful Alternatives (PCLHA)” unpacks PCLHA’s conditions, establishes its applicability at both the individual and collective level, shows that it is not wedded to any particular normative ethical theory, and situates it within the broader landscape of the philosophy of harm reduction. Section “Testing PCLHA: the ‘too Weak’ Objection and the Decisive Role of Functional Equivalence” tests PCLHA against the practices of eating conventional meat and manual driving, examining both existing alternatives (like plant-based options and public transit) and emerging technological alternatives (like cultivated meat and autonomous vehicles). This analysis justifies the sliding scale modification and highlights the importance of the functional equivalence condition in determining the principle’s applicability and plausibility. Section “Testing PCLHA: the ‘too Strong’ Objection” motivates the ‘too strong’ objection via the application of PCLHA to the cases of VR tourism (as an alternative to physical tourism) and sex bots (as an alternative to conventional recreational sex), before presenting the threshold of harm qualification as a response to this objection. Section “The Practical Utility of PCLHA” then counters the concern that PCLHA is practically inert by showing how the principle justifies mandates for existing less harmful alternatives in cases where voluntary adoption lags, and how it can be expanded to proactively incentivize the creation of such alternatives. The final section concludes.

## The Principle of Choosing Less Harmful Alternatives (PCLHA)

### Unpacking PCLHA

The principle of choosing less harmful alternatives is as follows:

*The Principle of Choosing Less Harmful Alternatives (PCLHA)*: It is morally wrong to continue to engage in a practice that causes harm if an affordable, accessible, functionally equivalent, less harmful alternative exists.

PCLHA does not inherently justify legal bans on the more harmful practice in question. One can maintain that a practice is morally wrong without believing that it should be illegal. To reiterate, *PCLHA outlines a sufficient condition for moral wrongness*,* not a necessary one.* While the satisfaction of the principle's conditions makes continuing with the more harmful practice wrong, many practices are morally wrong regardless of whether such alternatives exist. Dumping hazardous waste into rivers, for example, is wrong even if no functionally equivalent, less harmful alternative method of waste disposal is readily available.

PCLHA relates to numerous principles in normative ethics and public policy. It overlaps with the *Principle of Choosing the Lesser of Two Evils (PCLTE)* but has a different scope. PCLTE only applies to situations in which there are no net positive alternatives (only ‘evil’ ones), whereas PCLHA includes scenarios with such alternatives. PCLHA is narrower in scope than PCLTE, though, in that it is restricted to cases in which the alternatives are affordable, accessible, and functionally equivalent.^1^ Among normative ethical theories, PCLHA aligns most closely with utilitarianism, given its focus on the consequentialist consideration of harm reduction. However, PCLHA is not wedded to utilitarianism and is compatible with various normative ethical theories (King, 2020). Deontologists might view the principle as a means of fulfilling some fundamental moral duty (e.g., the duty to avoid causing unnecessary harm) (Hoffman, 2020), whereas virtue ethicists may construe the act of choosing less harmful alternatives as an expression of virtues like benevolence or compassion.

According to PCLHA, the alternative practice must be (A) affordable, (B) accessible, (C) functionally equivalent to, and (D) less harmful than the prevailing practice for there to be a moral imperative to make the switch. Regarding (A) and (B): What counts as “affordable” and “accessible” will vary based on contextual factors such as individual circumstances and geographic location. Regarding (D): PCLHA applies to the *least harmful* functionally equivalent alternative that is affordable and accessible at any given time. And it holds irrespective of the nature of the harms involved. Whether the harms from a practice are direct or indirect, probabilistic or guaranteed (Maheshwari, 2021), immediate or future-oriented (Parfit, 1984), what matters for PCLHA is simply whether an alternative practice reduces overall harm. Of course, determining *whether* alternative practices are less harmful than a prevailing practice, and *which* alternative is the least harmful, is often both theoretically and empirically challenging (precisely because the nature of harms differs across practices).^2^ Furthermore, some harms are genuinely incommensurate with one another (Norcross, 1997a).

### The Challenge of Functional Equivalence

Regarding (C): the ease of ascertaining functional equivalence varies considerably. In some instances, it may be clear that a less harmful alternative is functionally equivalent to the original practice that it seeks to replace. In others, it will be evident that a less harmful alternative is not functionally equivalent (e.g., replacing power tools with hand tools might reduce workplace accidents but fails to provide comparable functionality). In many (if not most) instances, however, what the criteria for functional equivalence are and whether these criteria are satisfied will be a contentious matter.

That ascertaining functional equivalence is difficult should not be surprising, given how contested the concept of a ‘function’ itself is across philosophy. In the philosophy of biology, for instance, there is considerable debate over the nature of ‘natural functions’ in biological entities (Millikan, 1989). In the philosophy of language debates arise over whether the meaning of words is synonymous with their function in language games (Wittgenstein, 1953), and in the philosophy of mind over whether mental states can be defined by their functional roles in cognitive systems (Block, 1980). This article is not concerned with biological functions, linguistic functions, or cognitive functions, but rather with the functions of human-made technologies and practices. Just as pain might be multiply realizable in different physical systems (e.g., human brains, octopuses’ nervous systems, or even AIs) so long as they play the same functional role, so too can any given practical function be multiply realizable in different practices or technologies. This is the pertinent notion of functional equivalence.

At a general level, it might be proposed that an alternative practice L is functionally equivalent to an original practice O if and only if L serves the same essential function as O. The problem is that what counts as an *essential* versus a *peripheral* function of a practice is highly disputable. For example, should objective measures of functionality (e.g., measurable performance metrics) be prioritized in deciding what counts as an ‘essential function’ over subjective measures (e.g., user satisfaction)? It is also unclear how stringent the functional equivalence condition should be (does it require exact parity? ) and what happens when there are trade-offs between functions, which is to say, when an alternative practice improves upon the original in one dimension but falls short in another. How does one adjudicate when and what trade-offs of functions are acceptable for functional equivalence? The challenges of navigating these tradeoffs resemble the challenges of resolving value conflicts in value sensitive design approaches to technology (Kozlovski, 2022).

I will clarify and make more tangible these difficulties with functional equivalence when applying PCLHA to the practices of conventional meat consumption and manual driving in the next section. Generally speaking, I do not think it is feasible to formulate a one-size-fits-all definition of ‘functional equivalence’ given that what functional equivalence means, and how its different dimensions are weighted, will depend upon the practice in question. This view aligns with work in the philosophy of technology which suggests that the function of artifacts is context-dependent rather than intrinsic to the artifact (Houkes & Vermaas, 2010).

Apart from tradeoffs between functions, in many cases tradeoffs will occur between functional equivalence and the other conditions built into PCLHA (i.e., harm reduction, affordability, and accessibility). For example, increasing the functionality of a practice can come at the cost of making it affordable, or of reducing its harm. When confronted with a tradeoff between functionality and harm reduction, society often values the former over the latter. As Norcross (1997a) highlights in his discussion of the so-called ‘lives for convenience’ principle, drastically lower speed limits on highways would reduce traffic accidents (satisfying the ‘harm reduction’ condition), but society is unwilling to adopt this alternative road practice because it would require forgoing substantial efficiency and convenience (i.e., the alternative practice fails to meet the ‘functional equivalence’ condition). While functional equivalence and harm reduction can come into tension in cases like this, they are not always at odds with one another, nor are they always fully conceptually distinct. The variable of safety, for instance, is both an aspect of harm reduction (unsafe alternatives will cause harm) and functional equivalence (an alternative that is significantly less safe than the original practice would fail to be functionally equivalent).

### Individual Versus Collective Applications of PCLHA

Having unpacked PCLHA’s conditions, an important remaining question is whether the principle operates at the collective level, the individual level, or both. The connection between an action X being morally wrong and there being an individual obligation to refrain from X is a topic of debate in applied ethics, especially in the context of collective action problems. For example, while it is morally wrong to continue to cause climate change, and there is a collective obligation to mitigate its effects, it is debatable whether and to what extent this collective obligation translates into an individual obligation to reduce one’s own carbon emissions (e.g., Kingston & Sinnott-Armstrong, 2018).

PCLHA clearly applies at the individual level because the conditions built into the principle set such a low bar for moral obligation. Once a less harmful alternative practice actually meets all of PCLHA’s conditions for a particular individual, that individual has a moral obligation to switch. Notably, individuals likely have moral obligations not just to adopt less harmful alternatives once they meet PCLHA’s conditions, but also to help bring about those conditions in the first place; for instance, by supporting policies and initiatives that would make alternatives more affordable and accessible. While PCLHA itself does not address such obligations, I briefly consider a proactive version of the principle that does in Sect. “The Practical Utility of PCLHA”. At the group level, PCLHA applies when its conditions are met at a collective’s operational scale. 

### PCLHA and the Philosophy of Harm Reduction

Before proceeding, it is worth situating PCLHA within the broader landscape of harm reduction approaches in applied ethics and public policy. Historically, the term ‘harm reduction’ is associated with a social and health services paradigm that arose in the mid-1980s in response to the HIV epidemic among injection drug users. The advent of the harm reduction model challenged the dominant paradigm of *prevalence reduction* that aimed to eliminate the prevalence of drug use, typically through criminalization. As Dea explains: “The new HR [Harm Reduction] model sought instead to target and reduce the attendant harms associated with drug use—in particular, the incidence of HIV and Hepatitis—through the use of needle exchange programs, safe injection sites, and similar” (Dea, 2020: 304). While PCLHA aligns with the general philosophy of harm reduction, it fundamentally differs from this traditional paradigm. Traditional harm reduction approaches target practices (a) that are intractable and persist regardless of their legal status, and (b) whose harms are exacerbated by their criminalization (e.g., sex workers feeling unable to go to the police when prostitution is outlawed). In contrast, PCLHA does not assume that the practices in question are intractable but, to the contrary, implies that such practices should be replaced by less harmful alternatives when they come along (often via emerging technologies). The principle also does not assume that criminalizing the prevailing practice will amplify its harms. PCLHA and ‘traditional harm reduction’ can thus be conceptualized as two subsets of the philosophy of harm reduction more generally.

Historically, the literature on harm reduction has focused on topics related to health and social policies, such as injection drug use (Lefor, 2019), physician aid-in-dying (Heilig & Jamison, 1996), kidney market legalization (Wilkinson, 2018), and E-cigarettes (Dawson & Verweij, 2017). This article fills a gap in the literature by advancing a novel harm reduction principle (PCLHA) and extending it across typically distinct domains of applied ethics. By employing PCLHA across these domains, the analysis implicitly suggests an argument for treating like cases alike, the idea being that if one upholds PCLHA, then they should apply the normative principle consistently across different spheres of life, from their food choices to their transportation practices.

## Testing PCLHA: the ‘too Weak’ Objection and the Decisive Role of Functional Equivalence

This section pressure-tests PCLHA through two case studies in food ethics and transportation ethics. First, I apply the principle to the practice of eating conventional meat, examining existing less harmful alternatives, such as veggie burgers, as well as the emerging technological alternative of cultivated meat. Then, I apply the principle to the practice of manual driving, examining the existing alternatives of public transportation and cycling as well as the emerging technological alternative of personal autonomous vehicles.^3^

### Food Ethics and PCLHA: from Conventional Meat to Cultivated Meat

The harms associated with conventional meat production are well documented, from the severe animal suffering within factory farming to environmental damage and negative health externalities (Singer, 1975). There is a powerful argument that factory farming is intrinsically wrong, and people already have a moral obligation to stop eating conventional meat and transition to less harmful alternatives. PCLHA provides a *complementary* line of argument that may persuade those unconvinced by claims about the intrinsic wrongness of conventional meat production. Assuming alternatives like veggie burgers are affordable and accessible, whether it is wrong not to switch to them according to PCLHA depends on whether they satisfy the functional equivalence condition. As discussed in the previous section, what ‘functional equivalence’ amounts to will differ, and be debatable, from case to case. Even if it is agreed that functional equivalence only requires replicating the ‘essential’ function of the original practice, there will be disagreement over what constitutes an essential versus peripheral function of conventional meat consumption. Is the essential function of this practice simply nutritional sustenance? If so, a veggie burger that matches the protein content and nutritional profile of a conventional meat burger would satisfy the functional equivalence condition. However, one could argue that the essential function of conventional meat consumption includes other elements like sensory experience. According to this broader interpretation, even a nutritionally identical plant-based alternative would fail the functional equivalence test if it cannot replicate the taste and texture of a meat burger. There is also the complication of tradeoffs between different functions. For example, if a plant-based alternative offers higher protein content and better digestibility than conventional meat but requires more preparation time and has a shorter shelf life, does it count as functionally equivalent?

While advocates of conventional meat consumption may argue that plant-based alternatives fail the functional equivalence test, proponents of these alternatives may accept this point and just take it to mean that PCLHA is insufficiently demanding and should thus be rejected as a normative principle. The underlying critique here is that PCLHA is ‘too weak’ because it lets people off the hook in cases of intolerable harm like factory farming on the technicality that less harmful alternatives are not perfectly functionally equivalent. An initial response to this objection is to reiterate that PCLHA represents only a sufficient condition for moral wrongness, not a necessary one. The advocate of PCLHA can thus agree that failing to switch to plant-based alternatives is morally wrong even if these alternatives are not functionally equivalent to, or as accessible or affordable as, conventional meat.

Even as just a sufficient condition for moral wrongness though, PCLHA can be challenged for treating functional equivalence as an all-or-nothing criterion. The solution is to modify the principle such that the degree of functional equivalence required by PCLHA varies inversely with the degree of harm at stake. Call this the ‘sliding scale’ modification:

*Sliding Scale Modification to PCLHA*: The degree of functional equivalence required by PCLHA varies inversely with (1) the severity of harm caused by the original practice and (2) the severity of harm reduction offered by the alternative.

This modification makes PCLHA more demanding in precisely those cases where the requirement for strict functional equivalence seems to let people off the hook too easily. It recognizes that, given the atrocities of factory farming and the amount of animal suffering at stake, continuing to choose conventional meat over vegetarian alternatives is morally wrong, even if these alternatives offer only partial functional equivalence. In contrast, for practices that cause relatively little harm, or for those where the reduction of harm offered by the alternative is relatively small, PCLHA would still require close to complete functional equivalence before it activates.

Consider now cultivated (or in vitro) meat, which is more decisively functionally equivalent to conventional meat than plant-based options like veggie burgers (Hopkins & Dacey, 2008). Cultivated meat is scientifically identical to conventional meat, the only difference being that it is grown in a lab rather than derived from living animals. In fact, cultivated meat has the potential to surpass conventional meat across multiple functional criteria like safety, nutrition, and taste. As Lo Sapio explains, “Cultivated meat could be manufactured to be nutritionally more balanced and healthier. Any ingredients are tracked, and production processes monitored. This can provide a greater quality control and hygiene. The resulting product could be infection, parasite, pathogen, and chemical contaminant-free” (2022, p. 28).

Of course, for PCLHA to hold for cultivated meat, it must meet all of the principle’s conditions, not just functional equivalence. The production of cultivated meat is, all things considered, significantly less harmful than the production of conventional meat. While the former’s production still involves some harms, such as the animal suffering related to the tissue extraction and the use of fetal bovine serum as a growth medium, these harms pale in comparison to the industrial-scale animal suffering intrinsic to factory farming.^4^ Cultivated meat also has the potential to reduce the environmental and health-related harms of conventional meat production through decreased land use, water consumption and, as mentioned above, reduced pathogen transmission risks.

In terms of affordability and accessibility: while the cultivated meat industry is growing rapidly, the technology is still too expensive and confronts numerous practical hurdles to its widespread implementation that must be overcome. There is, for example, massive cultural resistance to its adoption, as displayed by Florida and Alabama recently passing laws to ban it.^5^ For the purposes of this article, cultivated meat exemplifies how emerging technologies can be designed to more convincingly satisfy PCLHA’s functional equivalence condition compared to existing alternatives to harmful practices.

### Transportation Ethics and PCLHA: from Manual Driving to Autonomous Driving

Consider now PCLHA in the domain of transportation, specifically as it relates to the practice of manual driving. Unlike conventional meat production, the practice of manual driving is plausibly not *intrinsically* wrong. If it is wrong (or becomes wrong in the future), the wrong is *contingent* in nature, premised on the existence of less harmful alternatives. The harms of manual driving are unequivocal. Approximately 1.35 million road traffic fatalities and 20 to 50 million non-fatal injuries happen annually worldwide (Ratoff, 2022). Additionally, manually driven cars with internal combustion engines contribute to greenhouse gas emissions and air pollution. Even electric cars play a role in environmental degradation by adding to traffic congestion and noise and light pollution.

Numerous existing alternative transportation options like cycling and public transit (such as buses and subways) are clearly less harmful than manual driving in terms of both safety and environmental impact. Does PCLHA therefore imply that it is wrong to continue manually driving rather than switching to these alternatives? There is no universal answer to this question given that these alternative transportation options may satisfy all PCLHA’s conditions for some people in some geographic areas but not others. Public transportation may be affordable and accessible in cities but not rural areas. Likewise, cycling may be ‘functionally equivalent’ to manual driving for city folk with short commutes but not for those with longer commutes in suburban and rural areas.

Beyond acknowledging that PCLHA’s applicability is context-dependent, a central challenge here, as in the food ethics case, is settling on the criteria for functional equivalence. At minimum, it seems reasonable to say that a transportation alternative must be as safe as the average human driver across all driving conditions in order to be considered functionally equivalent to manual driving. Must the transportation alternative also be as fast as manual driving? What about as comfortable? And what happens when there are trade-offs between different functions such that a transportation alternative scores higher than manual driving along some dimensions like speed and convenience, but worse than manual driving along other dimensions like comfort and privacy? According to the sliding scale modification introduced in 3.1, the degree of functional equivalence required should vary with how grave the harms of manual driving are judged to be, and how much harm reduction the alternative transportation practices offer.

Personal autonomous vehicles are to personal manual driving in transportation ethics what cultivated meat is to conventional meat in food ethics. Just as cultivated meat is more functionally equivalent to conventional meat than existing plant-based alternatives, personal autonomous vehicles more closely replicate manual driving’s functionality than public transit or cycling. Unlike public transportation, personal autonomous vehicles preserve the privacy of personal manual driving, and unlike cycling, they afford comparable speed and reliability. From a harm reduction standpoint, autonomous vehicles can be algorithmically programmed to outperform human drivers across all driving conditions, including inevitable crash scenarios (Sui & Grève, 2024).^6^ While there is debate over precisely how safe autonomous vehicles will or could be in comparison to the average human driver, even a relatively conservative reduction of 10% fewer road fatalities per year equates to around 135,000 lives saved annually worldwide (Ratoff, 2022).

Autonomous vehicles also offer novel functionalities for the would-be driver, such as the ability to work or sleep during transit. That this newfound freedom to do other activities while commuting comes at the cost of the ‘freedom of manual control’ underscores the fact that functional tradeoffs exist even between practices as closely related as personal manual driving versus personal autonomous driving. Assuming autonomous driving is functionally equivalent (if not functionally superior) to its manual counterpart, there will still be no moral imperative to switch under PCLHA unless autonomous vehicles meet the affordability and accessibility conditions.^7^

I conclude this section with two points of synthesis. First, while emerging technologies like cultivated meat and autonomous vehicles may attain greater functional equivalence to the conventional practices they seek to replace than existing alternatives, they may also result in less harm reduction. The production of cultivated meat, as mentioned, still involves some animal suffering through processes like fetal bovine serum extraction, whereas plant-based alternatives avoid animal suffering altogether. Similarly, personal autonomous vehicles better replicate the convenience and reliability of manual driving compared to public transportation, but they may cause more environmental harm through continued individual vehicle production and usage than a widespread shift to mass transit systems.

Second, it is worth noting PCLHA’s applicability at both the individual and collective levels in these domains. In the manual driving case, while individuals have a moral obligation to switch to less harmful transit options when these options meet PCLHA’s conditions for them, collectives have parallel obligations (Collins & Lawford-Smith, 2016). Delivery companies, for example, are morally mandated under PCLHA to transition their fleets to electric vehicles once this less harmful alternative becomes viable at their scale of operation.^8^ Similarly, in the conventional meat case, individuals have a moral obligation to choose plant-based alternatives when they meet PCLHA’s conditions, but food services such as university dining systems and hospital cafeterias also have obligations to transition away from conventional meat if less harmful alternatives like veggie burgers and cultivated meat meet PCLHA’s conditions at their institutional level.

## Testing PCLHA: the ‘too Strong’ Objection

### Leisure Ethics and PCLHA: VR Tourism and Sex Bots

The previous section pressure-tested PCLHA by applying it to the practices of conventional meat consumption and manual driving. This analysis, among other things, illustrated that PCLHA can be criticized as insufficiently demanding because it does not require switching to less harmful alternatives when they do not perfectly match the functionality of the original practice, even when the original practice causes immense harm. In this section, I focus on a challenge to the principle from the opposite direction: the idea that PCLHA is too strong because it requires switching to less harmful alternatives in cases where this seems intuitively overreaching. Unlike factory farming (which is intrinsically wrong) and manual driving (which is plausibly a contingent wrong), the practices examined in this section, physical tourism and recreational sex, are arguably not wrong even if the alternatives of VR tourism and sex bots satisfy all of PCLHA’s conditions. Moreover, both physical tourism and recreational sex may be intractable in the traditional harm reduction sense, meaning they are likely to persist regardless of legislation or technological innovation. If so, this strengthens the ‘too strong’ objection by suggesting that PCLHA demands replacing practices that fundamentally resist replacement. 

To illustrate the objection, consider first how PCLHA would apply to physical tourism in the context of future virtual reality (VR) technology:

*VR Tourism and PCLHA*: It is morally wrong to partake in physical tourism in a world of affordable, functionally equivalent, accessible VR tourism experiences.

Long-distance leisure travel is already a controversial topic in applied ethics given its pronounced environmental impacts, among other reasons (Timmer & van der Deijl, 2023). VR tourism introduces a new dimension to this ethical debate. There already exist a plethora of VR tourism apps and experiences, such as the *Wander* app on the Meta Quest headset. The purported counterexample to PCLHA presupposes a futuristic version of VR technology that incorporates all sensory modalities and produces experiences that are phenomenologically identical to those in the physical world. Imagine Nozick’s (1974) experiment machine were the size and cost of an Xbox console and included a ‘whole earth’ inventory of VR tourism experiences. This is the vision.

Virtual tourism eliminates many of the harms associated with physical tourism, such as carbon emissions from air travel and physical wear and tear on natural and historical sites from tourist traffic. On the plausible assumption that VR tourism is overall less harmful than physical tourism, PCLHA leads to the counterintuitive, and arguably absurd, implication that it would be immoral to continue to participate in physical tourism in a world where experience-machine-quality VR alternatives are affordable, accessible, and functionally equivalent. This inverts Nozick’s insight entirely: rather than showing why we should not plug into the experience machine, PCLHA seems to mandate that we do exactly that. It tells would-be tourists to stay inside and plug into the matrix rather than go out into the world and touch grass.

An initial reply to this thought experiment is to simply deny that VR tourism could ever be experience-machine-quality in the sense of fully replicating the multisensory and contextually embedded nature of physical tourism. Just as Ramirez and LaBarge (2020) argue that there are insurmountable design problems (related to what they call ‘perspectival fidelity’ and ‘context-realism’) that make it technically impossible to create a phenomenologically identical simulation of the Bridge version of the trolley problem in VR, one might contend that essential elements of physical tourism experiences also cannot be wholly reproduced in VR.

Even if this is wrong though, and it is technologically possible to create VR tourism experiences that are phenomenologically identical to their physical counterparts, one might simply deny that *phenomenological equivalence* is synonymous with *functional equivalence*. That is, the fact that VR tourism experiences are phenomenologically identical to their physical counterparts does not necessarily mean they are functionally equivalent in the sense required by PCLHA. Perhaps *authenticity* (i.e., physical presence at actual places) itself constitutes an essential function of tourism.

While this dialectical move may disarm the VR tourism counterexample, the general critique that PCLHA is ‘too strong’ does not depend on this case alone. Consider a different version of the ‘Too Strong’ Objection:

*Sex Bots and PCLHA*: It is morally wrong to engage in recreational sex with other humans in a world of affordable, functionally equivalent, accessible sex bots.

This version of the objection imagines a lifelike version of sex bot technology that is imbued with artificial general intelligence and physically identical to humans. Notably, the application of PCLHA here does not concern sexual intercourse with other humans per se, but recreational sex specifically. Sexual intercourse of course has various functions beyond physical pleasure, such as procreation and emotional connection. The primary function of recreational sex specifically, however, is often mere physical pleasure (it is definitionally not procreation and typically is not expected to be deep emotional connection).

The assumption behind this version of the counterexample is that having intercourse with life-like sex bots is functionally equivalent to recreational sex with humans and less harmful, all things considered. Regarding harm reduction: the idea is that there would be a decrease in the likelihood of STDs, no chance of unwanted pregnancies or informed consent being violated (assuming that sex bots are not conscious moral patients), and plausibly fewer feelings of shame and embarrassment. PCLHA thus counterintuitively suggests that it would be morally wrong to continue to have recreational sex with other humans in a world of cheap, life-like sex bots.^9^

The proponent of PCLHA can respond to this version of the ‘Too Strong’ objection in the same way they responded to the VR version: by rejecting the notion that phenomenological equivalence is synonymous with functional equivalence. Even if intercourse with a sex bot is phenomenologically identical to recreational sex with a human, the two practices may not be functionally equivalent. Here, too, authenticity (i.e., genuine human connection) might be upheld to be an essential function of recreational sex.

### The Threshold of Harm Qualification

This authenticity-based defense successfully addresses the ‘Too Strong’ Objection to PCLHA, but it works only if one accepts that authenticity is an essential function of the relevant practices. A more robust response would avoid getting mired in debates over functional equivalence. I propose that such a response can be found in a limiting principle to PCLHA that takes the form of the following twofold qualification:

*Threshold of Harm Qualification to PCLHA*: PCLHA holds only if (1) the harm from the original practice is significant enough to warrant choosing an alternative, and (2) the alternative practice is significantly less harmful than the original.

If a practice X is not significantly harmful to begin with, then continuing with X is plausibly not morally wrong, even if a less harmful alternative Y exists. Conversely, even if X is significantly harmful, continuing with it may not be morally wrong if the alternative Y is not significantly less harmful.

Of course, determining when the original harm or the promised harm reduction is ‘significant enough’ to activate PCLHA will be subject to debate. Such a determination becomes even more complex when one considers that the threshold for what counts as ‘significant harm’ itself evolves as less harmful alternatives become widespread and normalized. For instance, once autonomous vehicles become common, the societal standards for what constitutes significant transportation-related harm will likely shift. While I cannot resolve these questions of harm measurability and shifting baselines here, the main point behind the ‘threshold of harm’ qualification is that PCLHA is intended to apply only in cases where both the harm from the original practice and the promised harm reduction of the alternative practice are substantial, not in trivial or borderline circumstances.

I submit that this limiting principle can be invoked to disarm both the VR tourism and sex bot variants of the Too Strong Objection. Turning first to whether the original practices in each case cause significant harm: physical tourism generates both harms (e.g., environmental degradation) and benefits (e.g., local economic revenue). Compared to conventional meat consumption and manual driving, the net harm of physical tourism is less clear and quantifiable. Similarly, the harms of conventional recreational sex may not be significant enough to warrant choosing a less harmful alternative.

Turning next to whether the alternatives offer significant harm reduction: VR tourism may not be significantly less harmful as a general practice than physical tourism. The widespread adoption of VR tourism as an alternative would bring about numerous harms, such as the psychological harms of social isolation and the economic harms of lost local tourist revenue. Likewise, the widespread adoption of sex bots might not be significantly less harmful than the contemporary prevalence of recreational sex. While sex bots will mitigate some of the harms of conventional recreational sex, they also threaten to increase existing harms associated with sexual objectification, social isolation, and misogynistic female body stereotypes, as well as introduce new harms related to negative symbolisms like the mimicking of sexual assault (Danaher, 2017).

The cases of VR tourism and sex bots thus plausibly fail to meet both conditions of the ‘threshold of harm’ limiting principle for PCLHA. In contrast, the harms from manual driving and conventional meat consumption are (1) significant enough to warrant less harmful alternatives, and (2) both existing alternatives (like public transportation and plant-based options) and emerging technological alternatives (like autonomous vehicles and cultivated meat) promise to be significantly less harmful than the original practices.

In light of the threshold of harm limiting principle, an objector to PCLHA might revise the VR tourism variant to a specific controversial case of physical tourism that clearly satisfies the requirements of the limiting principle. For instance, imagine there is an ongoing practice of physical tourism to an endangered ecosystem that is causing significant harm to the ecosystem, and as a less harmful alternative, a tech company develops a phenomenologically equivalent VR tourist experience of the ecosystem. This version of the VR thought experiment seemingly meets both pillars of the threshold of harm qualification to PCLHA because in this case, the original harm caused by the practice of physical tourism is undeniably significant, and the VR tourism alternative promises to eliminate such harms.

The problem with this modified version of the VR thought experiment, however, is that it no longer constitutes a counterexample or reductio ad absurdum to PCLHA. While it is counterintuitive to suggest that *physical tourism in general* is immoral due to the existence of affordable VR tourism, it seems reasonable to claim that it would be immoral to travel to a *specific endangered ecosystem*, especially given the availability of a phenomenologically equivalent VR reconstruction of that ecosystem.

## The Practical Utility of PCLHA

### When PCLHA Makes a Practical Difference

A final objection to PCLHA grants that the principle is theoretically sound but dismisses it as practically useless. The objection proceeds in two stages. First, PCLHA’s scope of applicability is limited by how notoriously difficult it is to achieve consensus on whether the principle’s conditions are satisfied. Second, as soon as consensus is reached that an alternative practice meets all of PCLHA’s conditions, the moral imperative to switch to the alternative becomes self-evident. The hard work consists in determining the criteria for functional equivalence and the methodology for measuring harm. PCLHA merely formalizes what is already obvious once this hard work is done. The principle is therefore either inapplicable or trivial. Call this the ‘No Practical Utility’ Objection.^10^

This objection correctly notes that adjudicating functional equivalence and measuring harm in any given case is where the hard work lies. Of course, PCLHA or something like it is the normative framework that gives this work its purpose. More substantively though, the No Practical Utility Objection overlooks historical cases in which it was clearcut that an alternative practice satisfied PCLHA’s conditions, yet the transition to the alternative did not happen voluntarily, often due to passive institutional inertia, active industry resistance, or sheer individual preference for the status quo. In these cases when voluntary adoption fails, PCLHA provides the normative justification for legal mandates. Furthermore, as emphasized throughout, the principle is specifically designed to put normative pressure on entrenched practices that are contingently wrong. PCLHA functions as a moral trigger point in these instances, specifying when technological progress converts a once-permissible practice into an impermissible one.

The clearest examples of PCLHA’s practical utility involve less harmful alternatives that are minor perturbations from the prevailing practice. Consider seat belts. The practices of driving with versus without a seat belt are nearly identical to the point that no reasonable person could deny that the two practices are functionally equivalent. There is also a clear discrepancy in harm between the practices. In the U.S. alone, driving with a seat belt saves roughly 15,000 lives per year (National Center for Statistics & Analysis, 2023) along with drastically reducing injury risk (Fouda Mbarga et al., 2018). And yet, although seat belts had become standard in cars by the late 1960s, only around 14% of drivers and passengers voluntarily used them as late as 1984 (National Highway Traffic Safety Administration, 2007). State laws were needed to compel people to start wearing seat belts.

To take an example from a different domain: lead-free paint alternatives like titanium dioxide were demonstrably functionally equivalent to lead paint for residential use by at least the 1950s (Laver, 1997), and evidence of childhood lead poisoning was conclusive, but residential use of lead paint did not stop in the U.S. until a federal mandate put an end to it in 1978 (Warren, 2001). These cases underscore the value of making implicit moral requirements (even self-evident ones) explicit. Such explication can shine a light on entrenched practices that violate those requirements and thereby help accelerate practical change.

### The Proactive Version of PCLHA

To be clear, these historical examples demonstrate PCLHA’s utility in cases where less harmful alternatives to a prevailing practice *already existed* (and clearly satisfied the principle’s conditions), but societal uptake lagged. The proponent of the No Practical Utility Objection may concede this point and shift to a different critique: that PCLHA does not inherently provide moral motivation to *create* less harmful alternatives to prevailing practices that do not yet exist. Strictly speaking, all the principle says is that *once* a less harmful alternative is affordable, functionally equivalent, and accessible, *then* it is morally wrong to continue the conventional practice. This logic does not in itself supply impetus to bring into existence such alternatives.

My response to this objection is twofold. First, the fact that PCLHA is devoid of moral motivation is not necessarily a problem for the principle as much as a clarification of its nature. PCLHA is not intended to be an all-encompassing normative principle; ethicists might endorse it alongside other principles that do the work of motivating the development of less harmful alternatives. Second, it is possible to revise PCLHA to proactively encourage the creation of such alternatives. For example:

*The Proactive Principle of Choosing Less Harmful Alternatives (PPCLHA)*: It is morally wrong to engage in a practice that causes harm if an affordable, accessible, functionally equivalent less harmful alternative exists (or could exist in a realistic close possible world).

Unlike PCLHA, PPCLHA carries a moral imperative to develop less harmful alternatives if they could be brought into existence in a realistic close possible world. The phrase ‘realistic close possible world’ refers to a hypothetical state of affairs that is not too far removed from the actual world along relevant dimensions (i.e., technologically, scientifically, economically, politically, socially). In the context of PPCLHA, this implies that the less harmful alternatives could feasibly be created and implemented at scale within a reasonable timeframe given the current state of affairs.

This proactive version of the principle addresses the question raised in the second section about whether individuals have obligations to help bring about the conditions under which PCLHA applies. According to PPCLHA, individuals do have such obligations, assuming these conditions (affordability, accessibility, functional equivalence) could be brought about in a realistic close possible world. While this makes PPCLHA more pragmatic than PCLHA, it also makes it more ethically demanding, setting a higher bar for moral obligations surrounding emerging technologies. Precisely how demanding PPCLHA is of course depends on how one interprets what counts as a ‘realistic close possible world’; for example, whether such proximity is judged based on current funding levels, political viability, technological feasibility, or some weighted combination of these and other factors. Together, PCLHA and PPCLHA delineate both the reactive and proactive facets of harm reduction ethics, designating when technological change makes existing practices impermissible, and when the absence of such change itself constitutes a moral failing.

## Conclusion

The purpose of this article has been to present and rigorously analyze the Principle of Choosing Less Harmful Alternatives (PCLHA). This principle represents a sufficient condition for when technological or social progress renders once-permissible practices impermissible. PCLHA is modest by design, owing to its numerous built-in conditions. While the principle is straightforward at first glance, applying it to concrete scenarios in food, transportation, and leisure ethics revealed that it can be contested for being both insufficiently demanding and overreaching. I addressed these critiques by adding two complementary revisions to the principle: the threshold of harm qualification and the sliding scale modification. Then, I demonstrated the principle’s practical utility.

Numerous aspects of the conceptual analysis presented here invite deeper development. In particular, there is a need for more detailed criteria for measuring functional equivalence, a more precise methodology for determining what counts as ‘significant harm’ in the threshold of harm qualification, and a more quantitative calculus for weighing degrees of functional equivalence against degrees of harm reduction in the sliding scale modification. Future work might draw on established threshold-based and gradational theories in normative ethics, such as threshold deontology (Brennan, 1995) and scalar utilitarianism (Norcross, 1997b), to inform these refinements. 

Future work should also account for the fact that many harms of emerging technologies are unpredictable and, sometimes, not even imaginable until they materialize after widespread deployment. Such uncertainty should be built into harm assessments when comparing prevailing practices with emerging technological alternatives. Further refining PCLHA in these ways will yield better guidance on how to navigate difficult tradeoffs. As discussed, emerging technologies like cultivated meat and personal autonomous vehicles may achieve greater functional equivalence to conventional practices than existing alternatives like plant-based diets and public transportation, but offer less harm reduction.

Finally, further work could explore the principle’s application in domains beyond those examined in this article. For instance, PCLHA holds that it is morally wrong to continue to conduct animal testing for medical research when less harmful alternatives such as computer modeling or in vitro testing meet the principle’s conditions. Likewise, in the domain of environmental ethics, the principle avers that it is morally wrong to continue relying on fossil fuels for energy production when less harmful renewable energy sources like solar and wind power satisfy the criteria.^11^ As emerging technologies continue to develop rapidly on multiple fronts, incorporating harm reduction principles like PCLHA into value sensitive design approaches and policymaking will become increasingly pressing. This article has laid some conceptual groundwork for how to systematically think about the ethics of harm reduction in emerging technologies.

## Data Availability

Not applicable.

## Footnotes

1 PCLHA differs in its approach to harm reduction from Mill’s classic Harm Principle. The latter concerns the legitimate use of state power to prevent harm to others (Mill, 1859; see Holtug, 2002 for a contemporary analysis), while the former focuses on when it is morally wrong to continue engaging in harmful practices when less harmful alternatives are available.

2 There are numerous challenges to quantifying the harms of a practice. On some views, risk itself is harmful, suggesting that a practice can cause harm via the imposition of pure risks (i.e. risks that do not manifest into material harm) (Maheshwari, 2021). Additionally, challenges in comparing harms arise when weighing current against future harms. For instance, in the context of animal research, the argument from avoidable harms against phase-out planning of such research states that “by restricting research, we may incur avoidable future harms and thus, while we may regret having to use animals in ways that harm them, for the sake of avoiding future harms we must not phase out animal research” (Müller, 2025, p. 111). PCLHA handles this argument by only requiring transitioning away from animal research when functionally equivalent alternatives like computer modeling and in vitro testing exist. In such cases, the future harms that Müller considers can be avoided through these alternatives rather than through continued animal testing.

3 The fields of transportation and food production both include a wide spectrum of existing and emerging alternatives with varying degrees of harm reduction. In the field of transportation, these alternatives range from walking, cycling, and scootering to carpooling, car-sharing services, and various forms of public transit, such as buses, light rail, and ferries. In the field of food production, alternatives span different farming methods (e.g., factory farming, free-range, organic), plant-based diets (e.g., vegan, vegetarian), and emerging technologies like cultivated meat, both with and without animal-derived growth mediums.

4 Some argue that promoting the development of cultivated meat may inadvertently (or advertently in some cases) cause harm by disincentivizing the growth of the plant-based meat industry and the uptake of vegan dietary lifestyles.

5 There are also numerous ethical worries that must be adjudicated, including the charges that cultivated meat perpetuates speciesism and violates animal dignity (Chauvet, 2018), sanctions the permissibility of cannibalism (i.e., cultivated human meat) (Milburn, 2016), causes a non-identity problem, and evokes a general feeling of repugnance (Kass, 1997).

6 Autonomous cars (even if they are not electric) also can cause less environmental harm by optimizing speed management (efficient acceleration and braking), improving traffic flow, and reducing light and noise pollution (Stone et al., 2020). However, because they do not require human drivers, they could conceivably increase traffic congestion by putting more cars on the road.

7 There are numerous ethical concerns surrounding autonomous vehicles that need to be adjudicated prior to their successful widespread implementation, such as how autonomous vehicles should be programmed to handle crash scenarios and more mundane situations (Himmelreich, 2018), how to solve human-robot coordination problems in mixed traffic scenarios (Nyholm & Smids, 2020), and the ‘right to drive’ question (Müller & Gogoll, 2020).

8 The displacement of delivery drivers of course raises additional ethical questions related to job automation that are beyond the scope of this article.

9 The contemporary debate in ethics of sex bots revolves around whether sex bots are morally permissible to begin with, not whether they should be preferred over recreational sex with other humans.

10 I would like to thank an anonymous reviewer for raising this objection.

11 Both the animal testing and energy production cases plausibly meet the functional equivalence criterion insofar as computer modeling and in vitro testing can yield similar scientific insights and results as animal testing, and renewable energy sources can provide the same energy output and reliability as fossil fuels.
